# The association between body mass index, exercise capacity, and health-related quality of life in heart transplant recipients

**DOI:** 10.3389/frtra.2024.1379695

**Published:** 2024-05-15

**Authors:** Margrethe Flesvig Holt, Stine Holmen, Katrine Rolid, Kristine V. Brautaset Englund, Charlotte M. Østby, Håvard Ravnestad, Arne K. Andreassen, Lars Gullestad, Einar Gude, Kaspar Broch

**Affiliations:** ^1^ Department of Cardiology, Oslo University Hospital, Rikshospitalet, Oslo, Norway; ^2^ Institute of Clinical Medicine, Faculty of Medicine, University of Oslo, Oslo, Norway; ^3^ Department of Medicine, Innlandet Hospital Trust, Hamar, Norway; ^4^ Department of Health and Public Sector, The Research Council of Norway, Oslo, Norway; ^5^ Division of Medicine, Akershus University Hospital, Lørenskog, Norway; ^6^ KG Jebsen Center for Cardiac Research, University of Oslo, Oslo, Norway

**Keywords:** heart transplant recipient, HRQoL (health-related quality of life), oxygen consumption (VO_2peak_), obesity, heart transplantation (HTx)

## Abstract

**Introduction:**

Pre-transplant obesity and weight gain after heart transplantation are both associated with increased risk of poor clinical outcomes. We aimed to assess the association between overweight or obesity, exercise capacity, and health-related quality of life in heart transplant recipients.

**Methods:**

This study is based on baseline data from the IronIC trial, in which we randomized 102 heart transplant recipients with iron deficiency to ferric derisomaltose or placebo. We performed cardio pulmonary exercise testing in all participants. To assess quality of life, we used the SF-36v2 questionnaire, using two sum scores: the physical component summary and the mental component summary. A minimal clinically important difference was defined as ≥2 and ≥3 for the physical and the mental component summary, respectively.

**Results:**

24/102 heart transplant recipients (24%) had a body mass index (BMI) ≥30 kg/m^2^. Peak oxygen consumption was 17.3 ± 4.6 ml/kg/min in the obese group vs. 24.7 ± 6.4 ml/kg/min in the group with a BMI <30 for a between-group difference of 7.4 (95% confidence interval 4.7–10.2) ml/kg/min: *p* < 0.001. The physical component summary score was on average 5.2 points lower in the patients with a body mass index ≥30 than in the lower weight group (*p* = 0.04).

**Conclusion:**

Almost a quarter of our heart transplant recipients in long-term follow-up had a BMI ≥30 kg/m^2^. These patients had substantially lower exercise capacity and lower quality of life in the physical domain.

## Introduction

Obesity prior to heart transplantation (HTx) and weight gain resulting in overweight or obesity after heart transplantation are associated with adverse cardiovascular events, allograft rejections and death (1–8). The effect of obesity and overweight on physical capacity and quality of life after heart transplantation has not been well studied.

Compared with the general population, heart transplant recipients have reduced physical capacity and reduced health-related quality of life (9–12). Exercise capacity improves substantially after heart transplantation (13). However, it stays lower than that of the general population, with peak oxygen consumption typically reported to range between 50%–70% of predicted values (12).

Quality of life increases substantially after heart transplantation but remains reduced compared with that of the general population (9, 14, 15). Several factors may contribute to reduced quality of life after heart transplantation, including the trauma associated with the pre-existing life-threatening heart failure and the subsequent surgery, as well as comorbidities and adverse effects of life-long treatment with immunosuppressants.

In the general population, the impact of overweight/obesity on health-related quality of life and physical capacity has been extensively studied. Obesity is associated with reduced functional mobility, fatigue, and depression (16–18). It is a well-known cause of reduced quality of life in the normal population (19). However, whether weight gain, overweight, and obesity are associated with health-related quality of life after heart transplantation remains largely unknown.

In this study we aimed to assess the association between obesity, defined as a body mass index (BMI) ≥30 kg/m^2^, exercise capacity, and health-related quality of life in contemporary heart transplant recipients.

## Method

### The IronIC trial

The randomised, controlled, double-blinded Intravenous Iron supplement for Iron deficiency in Cardiac transplant recipients (IronIC) trial was conducted at Oslo University Hospital, Rikshospitalet, the sole solid organ transplantation center in Norway, and has been reported in detail (20). In summary, 102 maintenance heart transplant recipients with iron deficiency and hemoglobin >100 g/L were randomized 1:1 to ferric derisomaltose or placebo (saline). The primary endpoint, peak oxygen consumption at six months after iron infusion, did not differ significantly between the groups (20). The trial was approved by the Regional Committee for Medical Research Ethics South-East Norway and conducted in compliance with the Declaration of Helsinki.

In this sub study of the Ironic trial, we aimed to assess the association between obesity or overweight and exercise capacity and health-related quality of life in heart transplant recipients. Obesity was defined as a body mass index (BMI) ≥30 kg/m^2^, in line with the cut-off values defined by the World Health Organization (21).

### Body composition

We assessed body composition using the InBody 770 bioelectrical impedance analyser. We registered weight and estimated total body water, total fat, skeletal muscle mass, and waist/hip ratio. Percentage body fat and percentage skeletal muscle mass was estimated by dividing fat mass and skeletal muscle mass, respectively, by total body weight and multiplying by 100.

### Peak oxygen consumption

We measured peak oxygen consumption on a treadmill with a breath-by-breath gas analyser as described previously (20). The test was continued until subjective fatigue was reached. We used a Borg scale value >18 points or a respiratory exchange ratio >1.05 indicative of an adequate maximal exercise test (22). The 2014 American College of Sports Medicine guidelines was used to determine predicted values (23). Patients who were unable to perform a treadmill test, performed the cardiopulmonary exercise test on an electrically braked bicycle ergometer.

### Hand grip strength

We measured hand grip strength using the Kern MAP hand-held dynamometer. The patients performed the test three times with their dominant hand. The highest value was recorded.

### Health-related quality of life

Health-related quality of life was examined using the SF-36v2 questionnaire, the EuroQol (EQ) 5D-3L and the EQ visual-analogue scale (VAS). The scores were converted into two norm-based sum scores: the physical component summary score and the mental component summary score. A minimal clinically important difference was defined as ≥2 for the physical component summary score, and ≥3 for the mental component summary score (24). The EQ-5D-3L questionnaire comprises five questions, each with three response alternatives addressing; mobility, self-care, usual activities, pain/discomfort, and anxiety/depression (25). We used a Swedish experience based value set to convert the scores into a summery index (26). The EQ VAS instructs the respondent to rate their overall health from 0 to 100 on a vertical visual analogue scale.

### Statistical analysis

All statistical analyses were performed using IBM SPSS Statistics for Windows, Version 28 (IBM Corporation). Data are expressed as means ± standard deviation, medians with interquartile range or numbers with percentages as appropriate. Chi-square test, Fisher's exact test, Mann-Whitney *U*-test, and *t*-test for independent samples were used as appropriate. A two-sided *p* < 0.05 was considered statistically significant.

## Results

### The IronIC trial

We enrolled 102 patients in the IronIC trial. Of the 102 participants, 24 patients (24%) were obese. Patient demographics by weight group (≥30 kg/m^2^ or <30 kg/m^2^) are presented in Table 1. Age, sex distribution, and time since transplantation were similar in the two groups. Use of beta blockers and loop diuretics was more frequent among patients with a BMI ≥30 kg/m^2^. There was a tendency towards ischemic reason being more and dilated cardiomyopathy being less frequent reasons for HTx in the patients with BMI ≥30 kg/m^2^. From approval for heart transplantation to inclusion in the IronIC trial, the patients with BMI ≥30 kg/m^2^ had gained 9.0 [−1.5 to 18.5] kg vs. 1.0 [−2.0 to 6.5] kg in the group with lower BMI (*p* = 0.007). For details see Supplementary Material.

**Table 1:** Patient characteristics.

	Body mass index ≥30 kg/m^2^ (*n* = 24)	Body mass index <30 kg/m^2^ (*n* = 78)	*p*-value
Age, years	55 [44–64]	56 [46–68]	0.75
Sex, female (%)	9 (38)	28 (36)	0.89
Systolic blood pressure, mmHg	131 ± 18	131 ± 17	0.90
Heart rate, beats per minute	89 ± 13	86 ± 12	0.41
NYHA functional class ≥III *n* (%)	2 (8)	3 (4)	0.34
Time since heart transplantation, years	8.3 [3.3–14.9]	7.3 [3.5–12.3]	0.64
Reason for HTx (%)			
Ischemic	12 (50)	16 (21)	0.08
DCMP	10 (42)	49 (63)	0.07
Other	2 (8)	13 (17)	0.51
Medication at inclusion *n* (%)			
Tacrolimus/Cyclosporine	16 (67)	56 (72)	0.62
Everolimus	10 (42)	25 (32)	0.46
Prednisolone	24 (100)	76 (97)	1.00
Mycophenolate	22 (92)	72 (92)	1.00
β-Blocker	13 (54)	24 (31)	0.05
ACE inhibitor/ARB	13 (54)	29 (37)	0.16
Loop diuretics	14 (58)	23 (29)	0.02
Biomarkers			
Hemoglobin, g/L	13.8 ± 1.5	13.7 ± 1.4	0.69
NT-proBNP, ng/L	546 [303–1,141]	313 [168–781]	0.08
Troponin T, ng/L	14 [8–28]	14 [8–24]	0.49
Creatinine, μmol/L	106 [86–139]	96 [81–111]	0.06
eGFR, ml/min/1.73 m^2^	61 ± 19	71 ± 20	0.02
Body composition			
Weight, kg	101 [92–106]	78 [68 86]	<0.001
Body mass index, kg/m^2^	31.8 [30.9–34.9]	25.6 [22.6–27.1]	<0.001
Body fat, % of total body weight	40 ± 9	27 ± 9	<0.001
Skeletal muscle mass, kg	34 ± 6.7	31 ± 5.9	0.04
Skeletal muscle mass, % of total body weight	33 ± 5	40 ± 5	<0.001
Waist/hip ratio	1.00 ± 0.11	0.90 ± 0.08	<0.001
Exercise/CPET			
Peak oxygen consumption, ml/kg/min	17.3 ± 4.6	24.7 ± 6.4	<0.001
% of expected peak oxygen consumption	50 ± 12	71 ± 17	<0.001
Respiratory exchange ratio	1.12 ± 0.08	1.13 ± 0.08	0.44
Absolute peak oxygen consumption, L/min	1.76 ± 0.49	1.89 ± 0.53	0.34
Hand grip strength			
Maximum hand grip strength, kg	34 [28–45]	34 [28–44]	0.97
Health-related quality of life			
SF-36 physical component summary score	42.4 ± 10.0	47.3 ± 9.5	0.04
SF-36 mental component summary score	52.3 ± 8.9	52.6 ± 7.5	0.88
EQ-5D3L, sum score	0.91 [0.83–0.93]	0.93 [0.86–0.97]	0.20
EQ VAS, score	76 ± 19	77 ± 16	0.81

Values are mean ± standard deviation, *n* (%), or median [interquartile range] as appropriate. DCMP, dilated cardiomyopathy; HTx, heart transplantation; NYHA, New York Heart Association; ACE, angiotensin-converting enzyme; ARB, angiotensin II receptor blocker; NT-proBNP, N-terminal pro-B-type natriuretic peptide; eGFR, estimated glomerular filtration rate; CPET, cardiopulmonary exercise testing; SF-36; short form-36; EQ-5D-3L, EuroQoL 5D - health-related quality of life three level version; EQ VAS, EuroQoL visual analogue scale.

### Body composition

**Figure 1:**
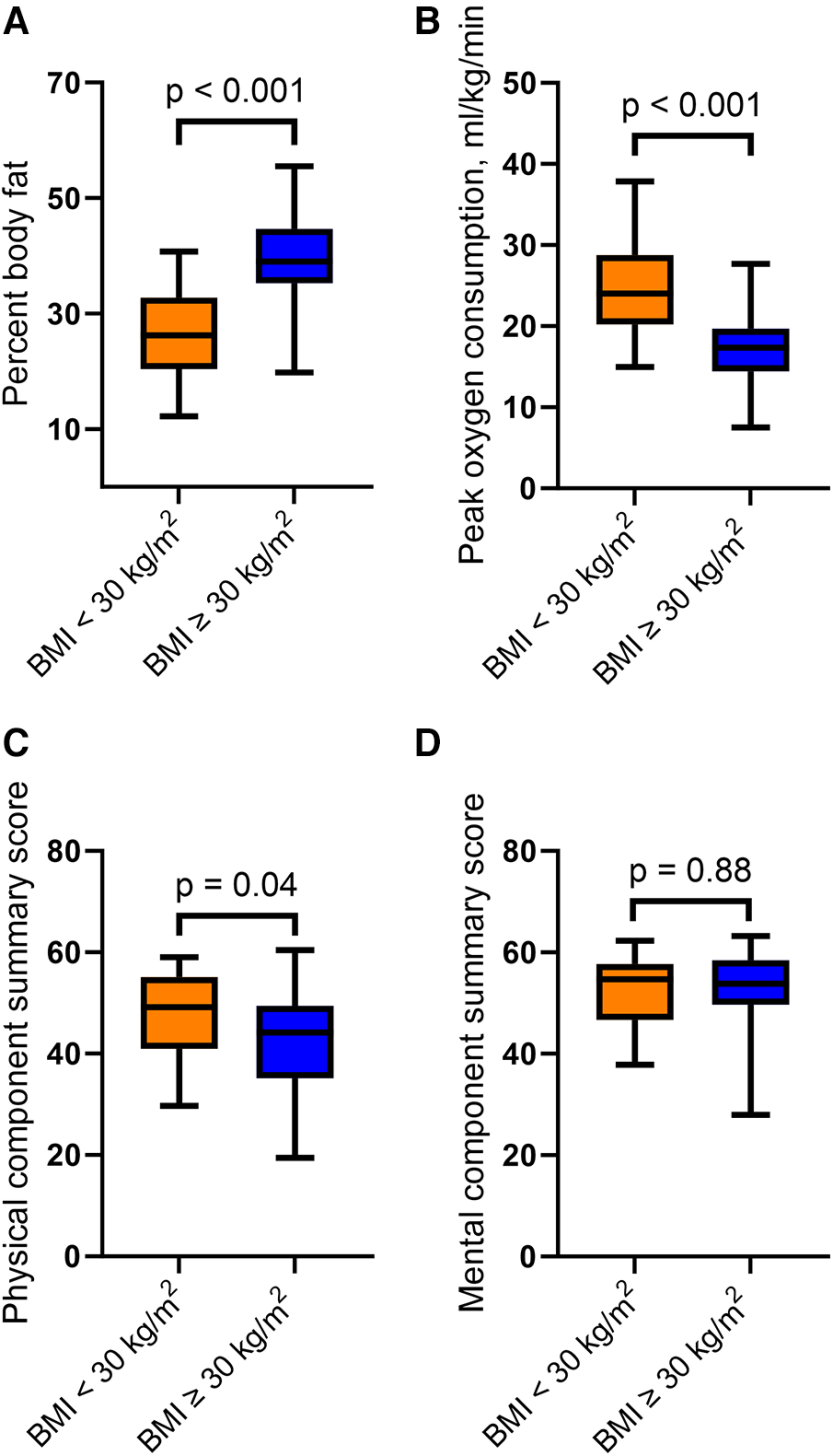
Difference in (**A**) percentage body fat, (**B**) peak oxygen consumption, (**C**) physical component summary and (**D**) mental component summary, stratified by BMI. *P*-values for the between group differences. Boxes: 25–75 percentiles; whiskers: 5–95 percentiles.

The body fat percentage was higher in the patients with a BMI ≥30 kg/m^2^ than in the group with lower BMI; 40 ± 9% vs. 27 ± 9%, *p* < 0.001 (Figure 1A and Table 1). The percentage of skeletal muscle mass of total body weight was lower in the obese patients compared with those with a BMI <30 kg/m^2^. Furthermore, patients with a BMI ≥30 kg/m^2^ had a significantly higher waist-hip ratio. For details, see Table 1.

### Peak oxygen consumption

The peak oxygen consumption was 17.3 ± 4.6 ml/kg/min in the patients with BMI ≥30 kg/m^2^ vs. 24.7 ± 6.4 ml/kg/min in patients with BMI <30 kg/m^2^, for a between-group difference of 7.4 (95% confidence interval 4.7–10.2) ml/kg/min; *p* < 0.001 (Figure 1B). The median respiratory exchange ratio was >1.05 and similar in the two groups, suggesting adequate maximal exercise tests in both groups (Table 1).

### Hand grip strength

There was no difference in hand grip strength between the patients with obesity and the patients with a BMI <30 kg/m^2^; (Table 1).

### Health-related quality of life

There was a statistically and clinically significant difference in the SF-36 physical component summary score between the two groups. The patients with BMI ≥30 kg/m^2^ had a mean physical component summary score of 42.4 ± 10.0 vs. 47.3 ± 9.5 in the patients with BMI <30 kg/m^2^; *p* = 0.04 (Figure 1C). On the other hand, there was no difference in the SF-36 mental component summary score between the two groups (Figure 1D). There were no differences in self-reported quality of life, as assessed by the EQ-5D and EQ VAS. For details, see Table 1.

## Discussion

Our results suggest that many Norwegian heart transplant recipients have overweight or obesity, resulting in part from obesity prior to transplantation and in part from weight gain after transplantation. Patients with a BMI ≥30 kg/m^2^ have substantially lower exercise capacity and lower health-related quality of life in the physical domain. Despite larger absolute muscle mass in the obese patients, hand grip strength and absolute oxygen uptake were not increased relative to those with a BMI <30 kg/m^2^.

Obesity prior to heart transplantation is associated with higher post-transplant mortality and with increased incidence of comorbidities, treated rejections, and cardiac allograft vasculopathy (6–8). The International Society for Heart Lung Transplantation (ISHLT) and the 2021 The European Society of Cardiology recommend a BMI <35 kg/m^2^ prior to listing for heart transplantation (22, 27). Excessive weight gain after heart transplantation is associated with an increased risk of cardiac allograft vasculopathy, non-fatal major adverse cardiovascular events, and allograft rejections (4, 5).

Health-related quality of life and exercise capacity increase after heart transplantation (9, 13–15). There is a positive correlation between health-related quality of life and exercise capacity in heart transplant recipients (10). While the negative effects of obesity on physical capacity and health-related quality of life are well documented in the general population (16–19), our study suggests that this also holds true for heart transplant recipients. There are already recommendations in place to reduce the risks associated with obesity in heart transplant candidates. However, our results suggest that weight control is important also after heart transplantation.

This study has some important limitations. The number of patients was limited, and the participants of the IronIC trial were required to have iron deficiency as defined in heart failure. However, our previous results suggest that this definition of iron deficiency is too liberal in maintenance heart transplant recipients, and that many of the participants were not truly iron deficient (20). We have no reason to believe that obesity is less prevalent in heart transplant recipients without iron deficiency, or that low iron stores would interact with the relationship between BMI and exercise capacity. Furthermore, our patients were included several years after heart transplantation. This make our data vulnerable for survival bias, and our study is not suitable to address survival.

In summary, obese heart transplant recipients had significantly diminished physical capacity and reduced health-related quality of life. However, we do not know whether normalization of BMI would be associated with improvements in these domains. Clinical trials targeting weight-loss in heart transplant recipients are warranted.

## Data Availability

The data analyzed in this study is subject to the following licenses/restrictions: Due to the sensitive nature of the data collected for this study, the material will not be made publicly available. Requests to access these datasets should be directed to mafhol@ous-hf.no.
