# Supplementary material for: The association between body mass index, exercise capacity, and health-related quality of life in heart transplant recipients
Source: Front Transplant. 2024 May 15;3:1379695. doi: 10.3389/frtra.2024.1379695 (PMC11235288; doi:10.3389/frtra.2024.1379695)
Supplement: Supplementary file 1 [file Datasheet1.pdf]

Supplementary Material.

Changes in weight and BMI from approval for heart transplantation to inclusion in the IronIC trial. Data on weight at approval for heart transplantation from were unobtainable from 7 patients.

Supplementary Table 1

	**Body mass index ≥ 30 kg/m^2 (n = 21)**	**Body mass index < 30 kg/m^2 (n = 74)**	**p-value**
Weight at approval for HTx, kg	91.7 ± 13.2	74.4 ± 13.3	<0.001
Weight at BL IronIC trial, kg	98.6 [91.5 – 106.3]	78.1 [68.2 – 85.7]	<0.001
Difference in weight from waitlist HTx to BL IronIC trial, kg	9.0 [-1.5 – 18.5]	1.0 [-2.0 – 6.5]	0.007
BMI approval for HTx, kg/m^2	30.3 ± 3.7	24.2 ± 3.3	<0.001

Abbreviations: HTx, heart transplantation; BL, baseline; BMI, body mass index. Values are mean ± standard deviation, median [IQR], independent sample t-test and Mann-Whitney U test used as appropriate.

Supplementary Table 2

	BMI approval for HTx, kg/m^2	BMI at BL IronIC trial, kg/m^2	Difference	p-value
**Body mass index < 30 kg/m^2 (n = 74)**	24.9 [22.1 – 26.3]	25.6 [22.6 – 27.1]	0.4 [-1.0 – 2.3]	0.048
**Body mass index ≥ 30 kg/m^2 (n = 21)**	29.9 [28.9 – 32.2]	31.8 [30.9 – 34.9]	3.2 [-1.1 – 6.2]	0.003

Abbreviations: HTx, heart transplantation; BL, baseline; BMI, body mass index. Values are median [IQR], P-values for paired samples waitlist vs IronIC study inclusion (Wilcoxon Signed Ranks Test).

The figure illustrates changes in BMI from approval for heart transplantation, at the time of transplantation, one year after transplantation and at inclusion in the IronIC trial.

Supplementary Figure 1

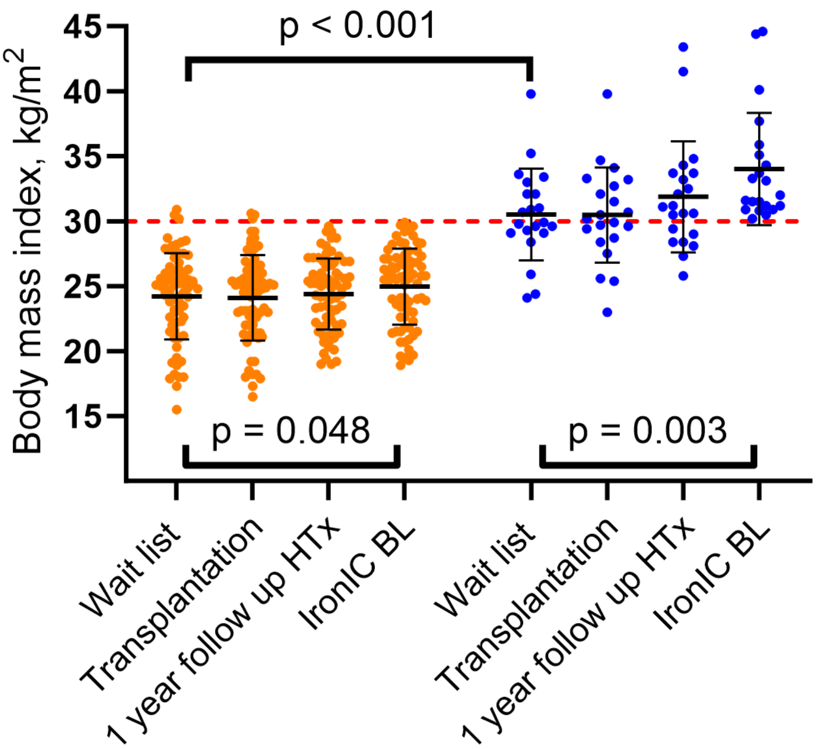

Column scatter graph showing the BMI for each participant (circles) at approval for transplantation (waitlist), time of transplantation (transplantation), the visit one year after transplantation (1 year follow-up HTx) and the time of inclusion in the IronIC trial (IronIC BL). The patients are stratified according to BMI at inclusion in the IronIC trial: BMI < 30 kg/m^2 (orange circles) and ≥ 30 kg/m^2 (blue circles). The red line is indicates BMI 30 kg/m^2. Mean values (horizontal line) with standard deviation (error bars) are shown.
